# Dysregulated autophagy contributes to the pathogenesis of enterovirus A71 infection

**DOI:** 10.1186/s13578-020-00503-2

**Published:** 2020-12-09

**Authors:** Chuanjie Zhang, Yawei Li, Jingfeng Li

**Affiliations:** 1 Department of Children Health Care, Wuhan Children’s Hospital (Wuhan Maternal and Child Healthcare Hospital), Tongji Medical College, Huazhong University of Science & Technology, Wuhan, Hubei People’s Republic of China; 2 Department of Health Services, Taihe Hospital, Hubei University of Medicine, Shiyan, Hubei People’s Republic of China; 3 Department of Pediatrics, Taihe Hospital, Hubei University of Medicine, Shiyan, 442000 Hubei People’s Republic of China

**Keywords:** Autophagy, Enterovirus A71 (EVA71), Nervous system injury, Pathogenesis, Hand, foot, and mouth disease (HFMD)

## Abstract

Enterovirus A71 (EVA71) infection continues to remain a vital threat to global public health, especially in the Asia–Pacific region. It is one of the most predominant pathogens that cause hand, foot, and mouth disease (HFMD), which occurs mainly in children below 5 years old. Although EVA71 prevalence has decreased sharply in China with the use of vaccines, epidemiological studies still indicate that EVA71 infection involves severe and even fatal HFMD cases. As a result, it remains more fundamental research into the pathogenesis of EVA71 as well as to develop specific anti-viral therapy. Autophagy is a conserved, self-degradation system that is critical for maintaining cellular homeostasis. It involves a variety of biological functions, such as development, cellular differentiation, nutritional starvation, and defense against pathogens. However, accumulating evidence has indicated that EVA71 induces autophagy and hijacks the process of autophagy for their optimal infection during the different stages of life cycle. This review provides a perspective on the emerging evidence that the “positive feedback” between autophagy induction and EVA71 infection, as well as its potential mechanisms. Furthermore, autophagy may be involved in EVA71-induced nervous system impairment through mediating intracranial viral spread and dysregulating host regulator involved self-damage. Autophagy is a promising therapeutic target in EVA71 infection.

## Introduction

Enterovirus A71 (EVA71) is a small, non-enveloped, icosahedral-shaped, positive-sense, single-stranded RNA virus belonging to the enterovirus, family *Picornaviridae*. EVA71 was first isolated from infants suffering from central nervous system (CNS) diseases in California in 1969 [1]. The first outbreak of EVA71 was reported in Bulgaria in 1975 [2], followed by rapid spreading worldwide. EVA71 infection has been reported to be prevalent in Asia–Pacific regions since the late 1990s [3].

EVA71 is one of the most common etiologic agents that cause hand, foot, and mouth disease (HFMD), which is a highly contagious disease around the world predominantly affecting children under the age of five [4]. HFMD has been classified as a notably infectious disease in category III since 2008 in China. During the past decade, a cumulative number of 20,537,199 HFMD cases, including 3667 deaths, have been reported by the Chinese Center for Disease Control and Prevention (China CDC). The symptoms of HFMD are usually mild. However, the neurological and cardiorespiratory complications associated with HFMD can be fatal [5], which is mainly associated with EVA71 infection [6, 7]. As a result, it is urgent to learn more about the pathogenesis of EVA71.

Autophagy is a conserved orchestrated process from yeast to human that maintains homeostasis by the degradation of misfolded proteins, damaged organelles, and/or intracellular pathogens [8]. Autophagy plays pivotal roles in protecting against neurodegenerative diseases, aging, cancer, and microbial infection [9]. However, increasing evidence has revealed the hijacking of autophagy and/or autophagic genes by EVA71 for its replication as well as neurological spread and impairment [10–14]. The corresponding mechanism remains elusive. This review discusses current studies on the interplay between autophagy activation and EVA71 infection, as well as the role of autophagy in EVA71 induced CNS injury, providing new therapeutic targets for EVA71 infection.

## The autophagy machinery in metazoans

**Fig. 1:**
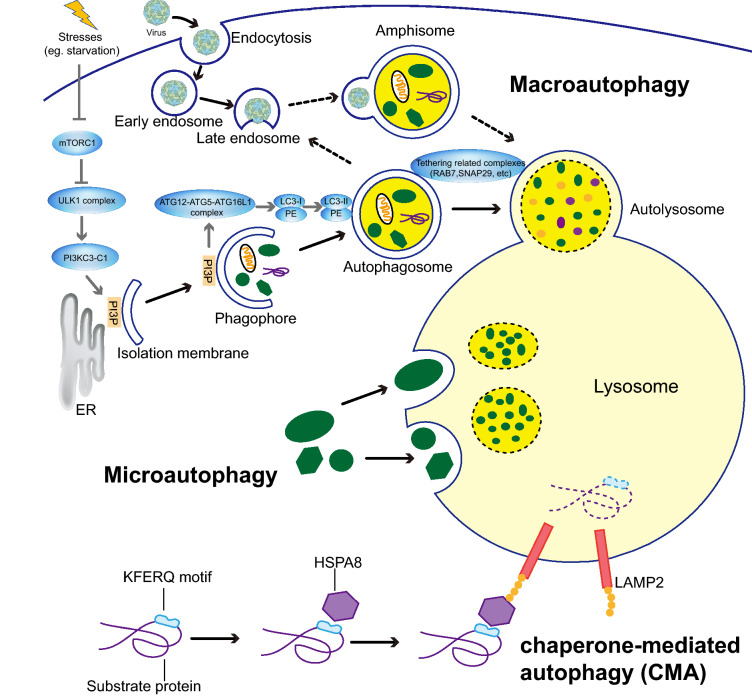
Overview of three types of autophagy in mammalian cells. During macroautophagy, cytosolic materials are first sequestered into a cup-shaped double-membrane structure, called phagophore, which elongates and matures into the double-membrane vesicle known as an autophagosome. The autophagosome fuses with the lysosome to form an autolysosome. Alternatively, the autophagosome fuses with the late endosome to form amphisome, which then fuses with the lysosome to form autolysosome. Gray arrows show mechanisms of autophagy. Autophagy is initiated by stresses (such as the mechanistic target of rapamycin kinase complex 1 (mTORC1)), followed by activation of the unc-51 like autophagy activating kinase 1 (ULK1) complex and phosphatidylinositol 3-kinase catalytic subunit type 3 complex I (PI3KC3-C1), which generates phosphatidylinositol-3-phosphate (PI3P). PI3P further recruits the ATG12-ATG5-ATG16L1 complex, which enhances the ubiquitin-like microtubule associated protein 1 light chain 3 (LC3)-I conjugates with PE to become the lipidated form LC3-II, contributing to autophagosome formation. Then a series of tethering related components are involved in the formation of autolysosome. Microautophagy involves the direct uptake of cytoplasmic materials through the invagination of the lysosomal membrane. Chaperone-mediated autophagy (CMA) can only degrade soluble proteins containing the KFERQ-like motif, which are recognized by the chaperone heat shock protein family A (Hsp70) member 8 (HSPA8), and directly across the lysosomal membrane by a receptor or translocon containing lysosomal associated membrane protein 2 (LAMP2) in the cytoplasm. During all three types of autophagy, the sequestered cargos are degraded by lysosomal hydrolases and recycled for the maintenance of cellular homeostasis

There are three major forms of autophagy in mammalian cells, namely, macroautophagy, microautophagy, and chaperone-mediated autophagy (CMA) (Fig. 1). All of them contribute to the overall intracellular autophagic activity [15, 16]. Among them, macroautophagy (to which further mentions of autophagy refer) is the major catabolic mechanism used by eukaryotes. In brief, autophagy induction leads to the recruitment of autophagy-related genes (ATGs) to the phagophore assembly site (PAS) to initiate and nucleate the phagophore (a cytoplasmic double-membrane cup-shaped structure). Then, the phagophore engulfs cytosolic organelles and proteins and elongates to form a double-membrane structure termed an autophagosome, which fuses with a lysosome to form an autolysosome that degrades the engulfing cargos. Alternatively, the autophagosome may fuse with the early and late endosome to form a vesicle known as an amphisome, which then fuses with the lysosome to form an autolysosome. The process of autolysosome formation and cargo degradation is also known as complete autophagic flux (Fig. 1) [17, 18].

Under the condition of nutrient-rich, host cell growth regulator MTOR (mechanistic target of rapamycin kinase) complex 1 (mTORC1) results in ULK1 (the unc-51 like autophagy activating kinase 1) and ATG13 phosphorylation, which negatively regulates ULK1 kinase activity and facilitates the dissociation of the ULK1 complex [19]. Turnover, when mTORC1 is inactivated under stresses, such as starvation, hypoxia, endoplasmic reticulum (ER) stress, and oxidative stress, the ULK1 complex is formed and subsequently triggers phagophore nucleation by phosphatidylinositol 3-kinase catalytic subunit type 3 (PIK3C3) complex I (PI3KC3-C1). The expression level of BECN1, one component of PI3KC3-C1, usually serves as a marker for early autophagy activation. The PI3KC3-C1 activates the production of phosphatidylinositol-3-phosphate (PI3P) at a specific ER structure omegasome, which further recruits the ATG12-ATG5-ATG16L1 complex. The protein complex further enhances the ubiquitin-like microtubule associated protein 1 light chain 3 (MAP1LC3-I/LC3)-I conjugate with membrane-resident phosphatidylethanolamine (PE) to become the lipidated form LC3-II [20], contributing to autophagosome membrane elongation and enclosure. Multiple factors, including tethering complexes (such as RabGTPase RAB7), core machinery synaptic-soluble *N*-ethylmaleimide-sensitive factor attachment receptor (SNARE), and members of the LC3/GABARAP family, are involved in the formation of the autolysosome [17, 21] (Fig. 1).

## The interplay between autophagy and EVA71 replication

**Fig. 2:**
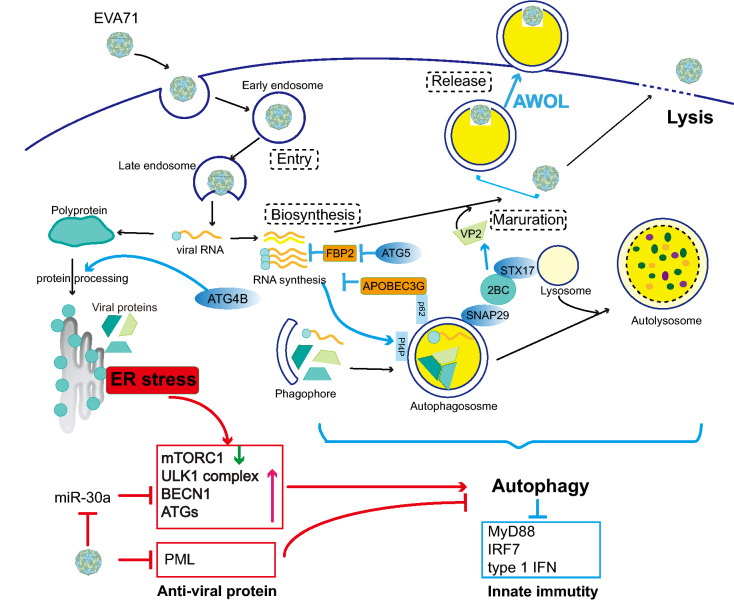
The interplay between EVA71 infection and autophagy. On one hand, EVA71 infection induces host autophagy activation through three major mechanisms as follows (red arrows): Firstly, unfolded or misfolded viral polypeptides during productive infection stimulate ER stress, which activates autophagy through inhibiting the activity of mTORC1 and activating ULK1 complex, BECN1 and ATGs. Secondly, EVA71 inhibits the generation of miRNA (has-miR-30a), which targets BECN1, leading to an increase in both mRNA and protein levels of BECN1. Thirdly, anti-viral protein PML (promyelocytic leukemia) is suppressed by EV71. The deficiency of PML is reported to trigger autophagy. On the other hand, increased autophagy activity may promote the EVA71 life cycle at different phases (blue arrows). The autophagic component ATG4B may involve in viral polyprotein processing. Autophagy may also provide membrane-bounded replication compartments for viral replication. ATG5 negatively regulates host anti-viral protein KH-type splicing regulatory protein (KHSRP, also known as FBP2), which involves in inhibiting EVA71 RNA translation. APOBEC3G, restricting 5′ UTR replication capacity of EVA71, co-localizes with p62 to the autophagic puncta and is degraded through the autophagy-lysosome pathway. Syntaxin-17 (STX17) and synaptosome associated protein 29 (SNAP29) interacting with EVA71 2BC promote viral maturation. Except for lysis, virions may release through the autophagosome-mediated exit without lysis (AWOL), which might involve in neurological infection. Besides, Toll-like receptors (TLR) signaling, represent the inhibition of innate immunity, is negatively regulated by EVA71 though autophagy (blue arrows)

The autophagy machinery plays a vital role in a multipronged defense against microbes [9]. However, several positive-sense, single-stranded RNA viruses (including Dengue virus and hepatitis C virus (HCV)), DNA viruses (such as Varicella-Zoster virus), and even bacteria (*Helicobacter pylori* or *Mycobacterium marinum*, for instance) have been demonstrated to activate autophagy to promote their replication/growth cycle at different stages [22–25]. Following Huang et al.’s report that the induction of autophagy in vitro and the formation of autophagosome-like vesicles in vivo after EVA71 infection benefits EVA71 replication [26], increasingly more studies have shown the hijacking of autophagy by EVA71 [10, 11, 13]. However, one study implied that increased autophagy levels contribute to the inhibition of EVA71 replication [27]. As a result, the specific trigger to regulate autophagy after EVA71 infection, as well as the specific influence of different stages of autophagy on the EVA71 replication cycle, remains to be investigated. In addition, recent studies have indicated that ER stress, dysregulated host microRNAs, and suppressed host anti-viral protein might contribute to the aberrant activation of autophagy by EVA71. In turn, activated autophagy may promote different stages of EVA71 life cycles and negatively regulate host innate immunity (Fig. 2).

### Autophagy is induced by EVA71 in vivo and in vitro

Increasing evidence has revealed that autophagy activity is increased in EVA71-infected mice models. Autophagosome-like vesicles are observed in mouse spinal neurons after oral infection of mouse-adapted EVA71 [26]. Suckling mice intracranially inoculated with EVA71 also show increased expression of LC3-II protein as well as the formation of LC3 aggregates and autophagosomes in infected brain tissues [28]. Furthermore, the colocalization of EVA71 structural protein VP1 or LC3 puncta and the endosome marker protein MPR are found in infected mice brain tissues, which represents the formation of amphisomes [28]. Thus, the various approaches involving EVA71 infection in mice models show an increased autophagic flux and the accumulation of autophagosomes.

Furthermore, EVA71 infection or exogenous overexpression of its structural viral proteins can also induce autophagy in vitro. Autophagosome- and autolysosome-like structures are found together with EVA71 particles in the cytoplasm of EVA71-infected SK-N-SH neuron cells [26]. EVA71 infection induces exogenous LC3 puncta and/or increases conversion of endogenous LC3-I to LC3-II in different cell lines [13, 14, 26, 29, 30]. Further, the colocalization of exogenous or endogenous LC3 dots and EVA71 VP1 protein is observed [13, 28, 31]. EVA71 infection also increases the expression of endogenous BECN1, while decreasing the protein level of p62 [13, 14, 29, 31]. These data provide convincing evidence regarding the induction of autophagy after EVA71 infection or overexpression of EVA71 structural proteins.

### Potential mechanisms underlying autophagy induction by EVA71

#### ER stress might be involved in EVA71-induced autophagy

ER stress, stimulated by unfolded or misfolded viral polypeptides synthesized during productive infection, tends to refold or degrade the unfolded/misfolded proteins to maintain homeostasis of the ER [32]. The ER protein quality control system achieves its function through two types of degradation pathways: ubiquitin–proteasome-mediated (type I) and autophagy-mediated (type II) ER-associated degradation (ERAD) of the unfolded protein response (UPR) [33]. Indeed, EVA71 replication is reported to induce ER stress, but to change the outcome of ER stress to assist viral replication through hijacking the ERAD component valosin-containing protein (VCP) [34, 35]. Although VCP is involved in both type I and type II ERAD [36], whether autophagy activation in EVA71-infected cells is associated with ER stress requires elucidation. Coxsackievirus B3 (CVB3) infection activates both ER stress and autophagy in HeLa cells: ER stress is induced and the ER stress sensors, PKR-like ER protein kinase (PERK), inositol-requiring protein-1 (IRE1), and activating transcription factor-6 (ATF6), are activated. The expression of MTOR and p-MTOR is decreased, while LC3 punctuation and the ratio of LC3-II/LC3-I are increased. Suppression of these three ER stress sensors using an inhibitor markedly decreases the ratio of LC3-II/LC3-I, suggesting the CVB3 may induce autophagy through activating ER stress [37]. In Li et al.’s study, the peripheral myelin protein 22 (PMP22), which is upregulated by EVA71 VP1 overexpression, was found to be associated with autophagy induction. Furthermore, inhibition of ER stress was found to suppress the expression of PMP22 significantly and thus to inhibit autophagy, implying that EVA71 might promote autophagy through the regulation of ER stress [30].

ER stress controls autophagy through several pathways [38]. First, sestrin-2 (SESN2) and DNA-damage-inducible transcript 4 (DDIT4), downstream of the ER stress, directly or indirectly (upregulated tribbles pseudokinase 3 (TRIB3) decreases akt murine thymoma viral oncogene homolog 1 (AKT1) phosphorylation) inhibit the kinase activity of MTORC1. Second, ER stress integrates all UPR branches at AMP-activated protein kinase (AMPK) and activates AMPK by phosphorylation and subsequently activates the ULK1 complex. Third, BECN1 and ATGs are regulated by ER stress: mitogen-activated protein kinase 8 (MAPK8) and death-associated protein kinase 1 (DAPK1) activation are implicated in the formation of PI3KC3-C1 through the phosphorylation and dissociation of BCL2 and BECN1, respectively. Transcription factors from all UPR sensors, such as Jun proto-oncogene (JUN, also known as c-Jun), X-box binding protein 1 (XBP1), activating transcription factor 4 (ATF4), DNA-damage-inducible transcript 3 (DDIT3), and ATF6, induce expression of BECN1 and other ATGs. Ca^2+^-dependent phosphorylation of protein kinase C, theta (PRKCQ) leads to its colocalization with LC3 on the elongating phagophore and facilitates autophagosome formation. The phosphorylated levels of MAPK1 proteins are increased in EVA71-infected GES-1 cells [39]. EVA71 infection also promotes c-Jun NH2-terminal kinase (JNK) phosphorylation, which might be related to autophagy activation [39, 40]. However, to date, little is known about the impact of ER stress on autophagy after EVA71 infection as well as its potential mechanism. Studies of this mechanism might provide potential targets for antiviral therapy against EVA71 (Fig. 2).

#### Host microRNAs targeted to the autophagy machinery component are down-regulated by EVA71

It is known that microRNAs modulate gene expression at the post-transcriptional level by binding to the 3′ untranslated region (UTR) region of mRNAs. Studies have reported that some microRNAs, including miR-146a and miR-296-5p, are associated with EVA71 infection and pathogenesis [41–43]. Some microRNAs have also been shown to regulate autophagy [44, 45]. EVA71 can also induce autophagy through the dysregulation of microRNAs in host cells. Fu et al. [13] determined that the relative expression level of has-miR-30a, which targets the 98-105 nucleotides in 3′UTR of BECN1, is reduced in EVA71-infected Vero and Hep2 cells. Subsequently, both mRNA and protein levels of BECN1 are induced, accompanied by an increase in the LC3-II/LC3-I ratio and a decrease in the p62 protein level, corresponding to increased autophagic activity [13]. More studies regarding aberrantly regulated microRNAs in EVA71-induced autophagy are expected to help uncover the mechanism of EVA71-related HFMD, as well as providing potential therapeutic targets.

#### EVA71 triggers autophagy through suppressing host anti-viral protein

Promyelocytic leukemia (PML) protein, the major component of PML-nuclear bodies (PML-NBs), plays vital roles in genome stability, programmed cell death, and antiviral activities [46]. Type I and type II interferon (IFN)-treatment-stimulated PML alternatively spliced isoforms are involved in the defense against several types of viruses, including EVA71 [47, 48]. EVA71 infection was found to reduce PML isoform III and IV expression as well as PML-NB formation mediated by EVA71 3C protein in HeLa cells. Further study showed that a deficiency in PML triggers autophagy (a decrease in p62, an increase in both LC3-II level and GFP-LC3 puncta-positive cells number) in vitro [47]. However, the mechanism underlying autophagy induction by EVA71 remains unclear and requires further investigation.

### Induced autophagy promotes EVA71 replication

Autophagy inhibitors treatment to block autophagosome formation decreases the extracellular virus titers and VP1 expression in EVA71-infected cell lines [13, 26, 31]. In contrast, after pretreatment with autophagy inducers (tamoxifen, rapamycin, or serum starvation), extracellular virus titers are markedly increased after EVA71 infection in SK-N-SH cells [26]. These results suggest that EVA71 replication rely on autophagy activity. Many Enteroviruses, such as poliovirus (PV), CVB3, enterovirus D68 (EVD68), and EVA71, utilize autophagy for their optimal infection. In response to different enteroviruses, autophagy produces membrane-bound replication compartments for viral RNA replication, virion maturation, or arrays autophagic vesicles for viral particle assembly or shedding, or promotes evasion from the host immune response [49–51]. This review also describes the details underlying the role of autophagy on different stages of EVA71 life cycles. However, more work is needed to investigate the detailed mechanism of how autophagy promotes EVA71 replication.

#### Autophagy core components facilitate the viral life cycle at different phases

*Viral biosynthesis* Studies have provided evidence that autophagy machinery plays vital roles in early and late stages of EVA71 biosynthesis events. For early events, the key component of the autophagy machinery autophagy related 4B cysteine peptidase (ATG4B) contributes to the maturation of early viral protein. ATG4B, a cysteine protease that cleaves pro-LC3 isoforms to form LC3-I, may function like EVA71 3C protein to hydrolyze the single polyprotein encoded by the EVA71 genome to generate early viral proteins to undergo the subsequent viral replication process [11, 52]. The silencing of ATG4B expression attenuates EVA71 RNA levels and VP1 expression levels in EVA71-infected RD, HEK293T, HeLa, and Vero cells [11].

During the late stage of viral biosynthesis, some enteroviruses depend on intracellular membranes for RNA replication [53, 54]. EVA71 3A protein can recruit phosphatidylinositol 4-kinase IIIβ (PI4KB) to RNA replication sites, which up-regulates phosphatidylinositol-4-phosphate (PI4P) to alter cellular membranes. Knockdown of PI4KB to reduce the synthesis of PI4P suppresses EVA71 RNA replication [55]. PI4P lipids may provide docking sites for PV and CVB3 RNA synthesis [54]. During autophagy, PI4P concentrates at the nucleation complex and recruits autophagy components to elongate the membrane structures [56]. PI4P generation on autophagosomes also promotes fusion with lysosomes [57]. However, the contribution of autophagy structures in EVA71 RNA replication still needs thorough investigation. BECN1 can interact with EVA71 3D protein (RNA-dependent RNA polymerase) to promote its replication (EV71 VP1 protein level) [58]. The 3D polymerase mainly promotes the extension of the EVA71 RNA chain [59], suggesting the possibility of viral RNA replication on autophagosome membranes. A previous study reported that autophagosome-like vesicles colocalize with PV RNA replication complexes [60]. Furthermore, PV double-strand RNA replicon colocalizes with LC3-positive structures at the late stage but not the early stage of infection. PV depends mainly on autophagosomes for maturation but not RNA replication [61, 62]. Although autophagy components colocalize with EVA71 RNA or polymerase, whether EVA71 RNA replication relies on autophagosome membranes is still undetermined.

Autophagy also triggers EVA71 replication through negatively regulating host anti-viral proteins. Host protein KH-type splicing regulatory protein (KHSRP, also known as FBP2), a component of the Dicer and Drosha complexes, binds to the EVA71 5′ UTR which contains an internal ribosomal entry site (IRES) and downregulates IRES activity by competing with other binding proteins, then negatively regulates EVA71 RNA translation [63, 64]. However, FBP2 is cleaved in EVA71-infected cells. Truncated FBP (FBP1-503) activates the IRES-driven translation of EVA71. Furthermore, the silencing of ATG5 to inhibit autophagosome formation significantly increases the intact form of FBP2, which implies that autophagy is involved in the cleavage of FBP2 and the following translation of EVA71 genome RNA [65]. Another host restriction factor, apolipoprotein B mRNA editing enzyme catalytic subunit 3G (APOBEC3G), is also inhibited by EVA71-activated autophagy. APOBEC3G, a member of the APOBEC3 family, possesses the antiviral activity to restrict many types of viruses, including EVA71 [66–69]. Normally, it restricts 5′ UTR replication capacity of EVA71 by competitively binding to its 5′ UTR with poly(C)-binding protein 1 (PCBP1), which is required for enteroviruses replication [66, 70, 71]. However, upon EVA71 infection, p62 and APOBEC3G co-localize to the autophagic puncta from the cytoplasm in the presence of EVA71 2C. Moreover, EVA71 2C protein induces APOBEC3G degradation through the autophagy-lysosome pathway [66].

*Viral maturation* EVA71 2BC non-structural protein precursor triggers autolysosome formation. Researchers observed that 2BC overexpression decreased the accumulation of p62 and increased the accumulation of late autophagic vacuoles 48 h after transfection. 2BC interacts with syntaxin-17 (STX17) and synaptosome associated protein 29 (SNAP29), which is associated with the fusion of autophagosomes to lysosomes. The knockdown of STX17 or SNAP29 was found to reduce the ratio of VP2/VP0 and EVA71 plaque formation [72]. Autophagosome-lysosome fusion inhibitor bafilomycin A1 treatment was also found to inhibit the generation of VP1 [73]. These studies suggest that autophagy machinery involved in autophagosome-lysosome fusion contributes to EVA71 maturation (Fig. 2).

Autophagosome fusion with lysosomes and degradation require an acidic environment [74]. The acidic compartment in the cell contributes to the maturation of some enteroviruses. Inhibitors of vesicle acidification do not impact PV RNA replication, but do impact the maturation of virions. NH_4_Cl treatment markedly increases VP0 abundance, indicating that acidification inhibition suppresses the cleavage of capsid protein to generate mature virus [62]. Prohibitin 2 directs EVA71 infection (intracellular EVA71 RNA and supernatants viral titers) as partially dependent on the acidification of autolysosomes [75]. However, the question remains of whether acidic vesicles directly affect EVA71 uncoating or maturation or both.

*Viral release* Non-enveloped viruses are usually released via rupture of infected cells. However, many enteroviruses, such as PV, CVB3, EVD68, and EVA71, can spread through the non-lytic pathway, including autophagosome-mediated exit without lysis (AWOL) [12, 76–79] (Fig. 2). For EVA71, AWOL is not only potentially involved with viral release but also potentially associated with CNS infection. Studies have suggested that EVA71 enters the CNS in mice via active retrograde axonal transport, which suggests that EVA71 replicates in skeletal muscles and infects motor neurons at neuromuscular junctions to reach the CNS [80–82]. During this process, virus particles may spread through AWOL. EVA71-infected neuron cells, including mouse motor neuron NSC-34 cells, human neural stem cells, and differentiated neuroblastoma IMR-32 cells, neither exhibit cytopathic effects nor undergo apoptosis. Instead, complete autophagy flux is markedly induced [12, 83]. Many EVA71-containing autophagic vesicles can be isolated from the culture supernatant of NSC-34 cells [12]. As a result, autophagic vesicles might be involved in the dissemination of EVA71 throughout the CNS, which ultimately leads to neurological symptoms in EVA71-infected patients. Although both autophagy and apoptosis were induced in EVA71-infected (Anhui-strain-infected) RD cells, the authors also reported that inhibition of apoptosis promotes autophagy activity [14]. Furthermore, inhibition of both autophagy and apoptosis decreases EVA71 viral particle release [14], implying that autophagy might promote viral release in EVA71-infected cells.

#### Induced autophagy represses innate immunity in response to EVA71 infection

Toll-like receptors (TLRs) act as molecular sentinels that recognize invading viral RNA and trigger host innate immune responses to eliminate the virus [84]. Selective activation of TLR7 inhibits EVA71 replication and increases the survival rate of ICR mice infected with the mouse-adapted EVA71 strain MP10 [85]. In reverse, EVA71 infection down-regulates TLR7 and its downstream products in 16HBE cells, including MYD88, interferon regulatory factor 7 (IRF7), and the secretion of type I IFN, markers for activation of innate immune responses. A further study found that after autophagy is suppressed by pretreating with 3-methyladenine (3-MA), EVA71 infection fails to inhibit the expression levels of TLR7-signaling-related molecules. In addition, viral titers and VP1 expression are down-regulated. These results suggest that autophagy facilitated EVA71 replication might be mediated by repression of innate immune responses [31].

### Speculation on the possible connection between autophagy and EVA71 receptor binding, viral entry, and uncoating

#### The possible role of EVA71 receptors on autophagy activity

Studies have reported many cell surface receptors for EVA71 infection, including scavenger receptor B2 (SCARB2), P-selection glycoprotein ligand 1 (PSGL-1), annexin II (Anx2), sialylated glycan, heparan sulfate proteoglycans (HSPG), dendritic cell-specific ICAM3-grabbing non-integrin, vimentin, nucleolin, fibronectin, and prohibitin [86]. Among them, the intrinsic lysosomal protein SCARB2 is the functional receptor mediating the attachment, internalization, and conformational changes at low pH [86]. Other molecules are known as “attachment receptors” that support viral attachment to the cell surface but cannot initiate uncoating. In addition, cyclophilin A (CypA) and tryptophanyl aminoacyl-tRNA synthetase (WARS) are involved in uncoating and an entry process respectively in the absence of SCARB2, although they are not defined as “attachment receptors” [87, 88].

Among these potential cellular receptors, vimentin [89] and nucleolin [90] are reported to inhibit autophagy activity. CyPA, triggered by hypoxia and infection [91, 92], leads to abnormal occurrence of autophagy [93]. Further study is needed to explore whether these potential receptors affect autophagy activity after EVA71 infection.

Amino acids are important environmental stimulants of autophagy activation [94]. The aminoacyl-tRNA synthetases are an essential enzyme family with 23 known members for amino acids synthesis [95]. These synthetases may participate in the regulation of autophagy activity. Indeed, tyrosyl-tRNA synthetase partially induces autophagy through up-regulating sirtuin 1 in pheochromocytoma PC12 cells treated with resveratrol (a natural polyphenol) [96]. Leucyl-tRNA synthetase activates mTORC1 by sensing intracellular leucine concentration and then binding to Rag GTPase to mediate amino acid signaling, which inhibits autophagy activity [97]. Glycyl-tRNA synthetase translocates to the nucleus to trigger NFκB1 upon treatment with methionine. Activated NFκB1 binds to the promoter of MTOR and activates MTOR signaling, which inhibits autophagy in bovine mammary epithelial cells [98]. However, the role of WARS, another aminoacyl-tRNA synthetase, in autophagy remains elusive. Multiple studies provide evidence that EVA71 infection triggers IFN production in different degrees in specific cells [99–101]. IFN-γ treatment increases WARS expression and translocates WARS to the plasma membrane, rendering semi-permissive and non-permissive cells susceptible to EVA71 [87]. WARS treatment further promotes inflammatory cytokines and type I IFN production [102], whereas type I IFN tends to induce autophagy [103]. It seems that WARS promotes autophagy after EVA71 infection. Nevertheless, how WARS, as a potential cellular receptor of EVA71, affects autophagy activity in EVA71 infected cells still needs elucidation.

Prohibitin 2, an inner mitochondrial membrane protein from the prohibitin protein family, is a mitophagy receptor mediating mitochondria for autophagic degradation [104]. A recent study reported that the C-terminus (aa 251–297) of EVA71 VP1 contributes to an increasing LC3-II/LC3-I ratio through interaction with prohibitin 2 [75], indicating that EVA71 may promote autophagy activity via prohibitin 2. In turn, prohibitin 2 directed EVA71 infection (intracellular EVA71 RNA and supernatants viral titers) in a manner partly dependent on the acidification of autolysosomes, which is involved in the complete autophagy induction [75]. However, the specific role of prohibitin 2 on the “positive feedback” between EVA71 replication and autophagy activity is still unknown.

#### Possible function of autophagy machinery on viral entry and uncoating

Enteroviruses usually enter cells through endocytosis mediated by clathrin, caveolae, or dynamin, etc. [105], followed by uncoating to release the viral genome for replication. Autophagy machinery may be involved in this process. RAB7, a member of the family of small GTPases involved in late endosomes and autophagosome maturation, regulates the trafficking of cargos along microtubules and their fusion with lysosome [106], and mediates Echovirus 7 movement to late endosomes and uncoating [107]. BECN1 induces late endosome maturation via interaction with UV radiation resistance-associated gene (UVRAG) [108]. ATG12 (a ubiquitin-like molecule)-ATG3 (an enzyme that mediates LC3 lipidation) conjugation promotes late endosome to lysosome trafficking [109]. However, whether autophagy participates in the internalization or uncoating of EVA71 remains in question.

## Autophagy is involved in EVA71-induced nervous system injury

Notably, 93% of laboratory-confirmed deaths due to HFMD between 2008 and 2012 in China were associated with EVA71 [7]. A recent observational cohort study from Colorado identified that EVA71 accounts for 58% of child cases of enterovirus-induced neurological disease [110]. EVA71 can occasionally cause severe neurological complications, including brainstem encephalitis, aseptic meningitis, and acute flaccid paralysis, which are considered to be a major factor in most fatal cases [111]. Brainstem encephalitis is the most common neurological problem among those complications, accounting for 58.8% (20/34) of neurological manifestations in a retrospective review [112]. It is also the most fatal neurological presentation because it can cause neurogenic pulmonary hemorrhage or edema that leads to death [113]. Some of these patients may develop long-term neurodevelopmental and psychiatric disorders even though they recover from this severe disease [114, 115]. Nevertheless, the associated mechanism is still not clear. Recent studies suggest that autophagy levels have essential roles in physiological neuronal processes, while impairment of autophagy leads to neurodegeneration [116]. EVA71 infection promotes autophagosome and amphisome formation in both neuron cell lines and mouse brain tissues [28]. Blocking EVA71-induced autophagy using 3-MA attenuates the disease symptoms and decreases the viral load in the brain tissues of the infected mice [28]. Autophagic vesicles may be involved in the spread of EVA71 to the CNS [12, 83]. These reports indicate that autophagy plays a vital role in EVA71-induced neurological injuries.

### The viral receptor may participate in neurological damage through autophagy

The expression of SCARB2 has been observed in almost all organs in humans, prominently so in neurons and lung pneumocytes [117]. SCARB2 expression is also detected in EVA71-antigen-positive neurons from patients who die of acute neurological disease [118]. Adult mice are not infected by EVA71. However, human SCARB2 transgenic mice exhibit paralytic diseases after EVA71 inoculation via intracerebral, intravenous, and intraperitoneal routes, which—like the symptoms observed in humans infected with EVA71—suggests that SCARB2 is essential to EVA71-induced neurological diseases [117]. However, the mechanism is still not clear. A study found that loss of SCARB2 function by mutation promotes the formation of lysosomes and autophagosomes. However, these autophagosomes contain partially degraded intracellular organelles, and some of them are in the process of exocytosis outside the cells, suggesting that SCARB2 might facilitate the fusion of lysosomes with autophagosomes [119]. Combined with the crucial role of SCARB2 in lysosomal biogenesis [120], SCARB2-regulated autophagy is assumed to participate in EVA71-induced neurological symptoms. However, direct evidence is still lacking. It is also hard to explain why the CNS is so susceptible to EVA71 infection even though SCARB2 is widely expressed in various human tissues.

HSPG are ubiquitous cell surface and extracellular matrix receptors, comprising repeating disaccharide units [121]. They usually interact with ligands through electrostatic interactions, such as EVA71 VP1 residues around the five-fold axis [122]. Natural mutant EVA71 VP1 BC loop (97R167G) variants acquire nerve cell tropism, which leads to an increased ability to bind HSPG and a relatively high expression level of HSPG in neurons and glial cells, although the infection still depends on SCARB2 [123]. A study in *Drosophila* found that HSPG biosynthesis is critical for normal assembly of postsynaptic membrane specializations through regulating autophagy activity [124]. It would be interesting to explore whether autophagy is involved in the neuron tropism of specific EVA71 mutant mediated by HSPG.

### Autophagy may indirectly mediate neuronophagia

A pathological study on 14 autopsied patients of EVA71 infection showed neuronophagia by neuroglia in brainstem neurons [125]. Another study reported that up-regulated cell-surface-exposed calreticulin may act as a phagocytic signal, which promotes viable neuron phagocytosis by microglia to induce neuronal death [126]. In vitro experiments have shown that exogenous overexpression of EVA71 capsid proteins, especially VP1, causes neuron injury not through inducing apoptosis directly but through activating ER stress, which might subsequently activate autophagy. Moreover, VP1-induced ER stress and autophagy up-regulate the expression of cell surface-exposed calreticulin [125]. These results suggest that autophagy indirectly mediates neuronophagia.

## Autophagy pathway is a potential therapeutic target for EVA71 infection

No specific antiviral treatment is available for EVA71 infection. The clinical manifestation in most cases is mild and self-limiting. Patients with neurologic or other severe illnesses need to receive supportive treatment. The first inactivated EVA71 whole virus vaccine for preventing severe HFMD was approved by the China Food and Drug Administration (CFDA) in Dec 2015 [127]. A longitudinal surveillance study showed that the average incidence rate of EVA71-induced HFMD in 2017–2018 was 60% lower than predicted [128]. However, HFMD remains a severe public health threat in Asia and the Pacific regions. It is urgent to obtain approval for effective drugs specific to EVA71. Great efforts have been devoted to the development of antiviral strategies, including passive immunization, immune modulators, anti-inflammation treatments, and various compounds [129]. The autophagy pathway is a potential therapeutic target of some natural compounds in EVA71 therapy.

A derivation of alkaloid component lycorine LY55, which is isolated from the bulbs of lycolium, has good activity against EVA71 replication in vitro and ICR mice. LY-55 treatment decreases body weight loss, as well as the protein level of EVA71 VP1 in infected mice. However, LY-55 is viricidal not directly but through downregulating autophagy (decreased LC3-II, while increased p62) by reduced JNK phosphorylation. Further study has shown that LY-55 and 3-MA synergistically inhibit EVA71 replication, suggesting that autophagy is a potential therapeutic target for the treatment of EVA71 infection [40].

Another natural compound is resveratrol, a naturally-occurring polyphenol first isolated from white hellebore. Resveratrol has a variety of biological functions, such as anti-oxidant, anti-inflammatory, and anti-tumorigenic effects; cardiovascular protection; and antiviral effects [130]. Researchers have investigated whether resveratrol treatment significantly decreases the expression of EVA71 VP1 in EVA71-infected- RD cells [131]. Resveratrol-loaded nanoparticle (RES-NP) treatment down-regulates the autophagy activation and mitigates the damage of RD cells by EVA71, as well as inhibiting the secretion of inflammatory factors, including interleukin (IL)-6, IL-8, and tumor necrosis factor-α elicited by EVA71 infection. Inhibition of autophagy abolishes the antiviral and anti-inflammatory effects of RES-NPs on EVA71-infected RD cells, implying that autophagy plays a pivotal role in RES-NPs inhibited EVA71 replication [131].

Berberine, an isoquinoline alkaloid isolated from several herbal substances, is commonly used for its antidiabetic, anticancer, and antimicrobial activity. Its metabolites also contribute to a series of pharmacological effects [132]. Studies have shown that both berberine and its derivative 2d (a compound introduced an alkylation substituent at the 9-position) inhibit the expression of VP1 and EVA71 replication in Vero cells. Further study found that berberine and compound 2d treatment decrease the expression of LC3-II. Berberine also increases the amount of p62 in EVA71-infected Vero cells. These results suggest that berberine and compound 2d attenuate EVA71-induced autophagy. Moreover, the phosphorylation level of AKT is increased, while those levels of JNK and PI3KIII are reduced at the presence of berberine and compound 2d, suggesting that berberine and compound 2d might suppress EVA71-induced autophagy through activating AKT and inhibiting the phosphorylation of JNK and PI3KIII [133, 134]. However, the mechanisms still need further study.

Saikosaponin D (SsD), one of the active components of *Bupleurum falcatum*, which is used to control infectious diseases [135], was shown to suppress EVA71 infection by inhibiting autophagy [73]. SsD treatment can delay the endosomal-lysosomal pathway and inhibit the fusion of autophagosomes and lysosomes through RAB5, suggesting that SsD is a late-stage autophagy inhibitor. SsD exposure also reduces the EVA71 positive RNA strand and VP1 protein level. Inhibiting autophagy at an early stage markedly suppresses the EVA71 VP1 protein level and vice versa. SsD treatment fails to further increase the effect of ATG5 on EVA71 infection. Treatment with the MTOR inhibitor Torin-1 dose not affect the effect of SsD on EVA71 either, indicating that SsD potentially prevents EVA71 infection through inhibiting late-stage autophagy [73]. However, more studies should be conducted on the effect of SsD on EVA71 infection through late-stage autophagy, and on the specific stage of the EVA71 life cycle.

## Conclusion

EVA71, like other enteroviruses, triggers the autophagy pathway and hijacks autophagy and/or autophagic genes for their life cycle. More studies are needed to investigate the details of different stages of autophagy used for the specific life cycle of EVA71. For example, although EVA71 particles are observed together with autophagosome- and autolysosome-like structures or VP1 protein are found colocalized with LC3 dots, no report has shown viral RNA or replication intermediate or early protein together with autophagosomes or autolysosomes. In addition, autophagy may directly mediate the spread of EVA71 to the CNS and replication in neuron cells or indirectly mediate neuronophagia via regulating host factors after EVA71 infection. However, the details of the susceptibility of EVA71 in the CNS are still not clear. Further study regarding the interplay between autophagy and viral replication, as well as autophagy in EVA71-induced neurological diseases, might provide a novel therapeutic strategy for EVA71 infection.

## Data Availability

Not applicable.

## Abbreviations

EVA71: Enterovirus A71
CNS: Central nervous system
HFMD: Hand, foot, and mouth disease
China CDC: Chinese Center for Disease Control and Prevention
CMA: Chaperone-mediated autophagy
ATGs: Autophagy-related genes
PAS: Phagophore assembly site
MTOR: Mechanistic target of rapamycin kinase
mTORC1: MTOR complex 1
ULK1: Unc-51 like autophagy activating kinase 1
ER: Endoplasmic reticulum
PIK3C3: Phosphatidylinositol 3-kinase catalytic subunit type 3
PI3KC3-C1: Phosphatidylinositol 3-kinase catalytic subunit type 3 (PIK3C3) complex I
PI3P: Phosphatidylinositol-3-phosphate
MAP1LC3: Microtubule associated protein 1 light chain 3-I
PE: Phosphatidylethanolamine
SNARE: Synaptic-soluble N-ethylmaleimide-sensitive factor attachment receptor
HCV: Hepatitis C virus
ERAD: ER-associated degradation
UPR: Unfolded protein response
VCP: Valosin containing protein
CVB3: Coxsackievirus B3
PERK: PKR-like ER protein kinase
IRE1: Inositol-requiring protein-1
ATF6: Activating transcription factor-6
PMP22: Peripheral myelin protein 22
SESN2: Sestrin-2
DDIT4: DNA-damage-inducible transcript 4
TRIB3: Tribbles pseudokinase 3
AKT1: Akt murine thymoma viral oncogene homolog 1
AMPK: AMP-activated protein kinase
MAPK8: Mitogen-activated protein kinase 8
DAPK1: Death-associated protein kinase 1
JUN: Jun proto-oncogene
XBP1: X-box binding protein 1
ATF4: Activating transcription factor 4
DDIT3: DNA-damage-inducible transcript 3
PRKCQ: Protein kinase C
JNK: c-Jun NH2-terminal kinase
UTR: Untranslated region
PML: Promyelocytic leukemia
PML-NB: PML-nuclear bodies
IFN: Interferon
PV: Poliovirus
EVD68: Enterovirus D68
ATG4B: Autophagy related 4B cysteine peptidase
PI4KB: Phosphatidylinositol 4-kinase IIIβ
PI4P: Phosphatidylinositol-4-phosphate
KHSRP: KH-type splicing regulatory protein
IRES: Internal ribosomal entry site
APOBEC3G: Apolipoprotein B mRNA editing enzyme catalytic subunit 3G
PCBP1: Poly(C)-binding protein 1
STX17: Syntaxin-17
SNAP29: Synaptosome associated protein 29
AWOL: Autophagosome-mediated exit without lysis
TLRs: Toll-like receptors
IRF7: Interferon regulatory factor 7
3-MA: 3-Methyladenine
SCARB2: Scavenger receptor B2
PSGL-1: P-selection glycoprotein ligand 1
Anx2: Annexin II
HSPG: Heparan sulfate proteoglycans
CypA: Cyclophilin A
WARS: Tryptophanyl aminoacyl-tRNA synthetase
UVRAG: UV radiation resistance-associated gene
CFDA: China Food and Drug Administration
RES-NPs: Resveratrol-loaded nanoparticles
IL: Interleukin
SsD: Saikosaponin D
